# Type of Preservation Solution, UW or HTK, Has an Impact on the Incidence of Biliary Stricture following Liver Transplantation: A Retrospective Study

**DOI:** 10.1155/2019/8150736

**Published:** 2019-12-21

**Authors:** Rojbin Karakoyun, Antonio Romano, Johan Nordström, Bo-Göran Ericzon, Greg Nowak

**Affiliations:** Division of Transplantation Surgery, CLINTEC, Karolinska Institutet, Karolinska University Hospital Huddinge, Stockholm, Sweden

## Abstract

Organ preservation plays a crucial role in the outcome following solid organ transplantation. The aim of this study was to perform a retrospective outcome analysis following liver transplantation using histidine tryptophan ketoglutarate (HTK) or the University of Wisconsin (UW) solutions for liver graft preservation. We retrospectively reviewed data on adult patients who were liver-transplanted at Karolinska University Hospital between 2007 and 2015. There was evaluation of donor and recipient characteristics, pre- and post-transplant blood chemistry tests, biliary and vascular complications, graft dysfunction and nonfunction, and patient and graft survivals. A total of 433 patients were included in the analyses, with 230 and 203 patients having received livers preserved with HTK and UW, respectively. Mean follow-up was 45 ± 29 months for the HTK group and 42.4 ± 26 for the UW group. There was no difference between the two groups either in terms of patient and graft survival, or of results of postoperative blood chemistry, or incidence of arterial complications, early allograft dysfunction, or primary graft nonfunction. However, the incidence of biliary stricture was higher in the UW group (22.7%) versus the HTK group (13.5%; *p*=0.013). Use of UW and HTK preservation solution in liver transplantation has no impact on patient and graft survival. However, use of HTK solution results in a lower incidence of posttransplant biliary stricture.

## 1. Introduction

Organ preservation plays an important role in solid organ transplantation [1]. The University of Wisconsin (UW) solution has been the most widely used for liver transplantation for some decades, and it is still regarded as the gold standard for liver preservation ever since 1987 [2–4]. However, it has been shown that hydroxyethyl starch included in UW solution induces red blood cell aggregation, which could promote occlusion and incomplete washout of blood from donor organs during cold perfusion [5, 6]. One alternative to UW solution is histidine tryptophan ketoglutarate (HTK) solution. Potential advantages of using HTK in liver preservation are its lower viscosity, low potassium content, and lower cost [7]. Low viscosity of the preservation solution may provide a better initial flush of the liver, more rapid cooling, and improved washout of blood during organ procurement. The first clinical results of use of HTK in liver transplantation were reported in 1990 [8]. The first randomised comparison between UW and HTK solution was reported 20 years ago, and since then, HTK has been found to be equivalent to UW solution where cold ischemia times do not exceed 15 h [8–10]. Recently, two large registry studies have shown better graft and patient survival following preservation of the liver graft using UW solutions [11, 12].

The aim of this study was to perform a retrospective outcome analysis following use of HTK and UW solutions in adult deceased donor liver transplantation (DDLT).

## 2. Material and Methods

We analysed the data from all liver transplantations performed at the Karolinska University Hospital in Stockholm between May 2007 and June 2015. Patients above 18 years of age at the time of transplantation were included. Patients transplanted with grafts from living donors, domino donors, multivisceral organ transplantation, patients with portacaval transposition, and intraoperative death were excluded from the study. Moreover, in patients receiving a second graft within 3 years of the first transplant, the second transplant was excluded from the analysis.

During the study period, UW and HTK were used for liver preservation “quasi”-randomly. Livers were obtained from two donor centers. With the existing collaboration between the two centers, teams from each of the centers were responsible for harvesting organs within the same donor area on alternate weeks. We included also livers harvested outside our standard donor area but still within Scandinavian countries. One team used only UW as a preservation solution, while the other team used exclusively HTK. Members of both procurement teams are equally trained in abdominal organ procurement and represent the same level of surgical skills including random involvement of liver transplant surgeon in the procurement team.

Rapid perfusion technique with only aorta perfusion was used. Organs were harvested separately after dissection *in situ*. Volumes of used perfusion fluid were recorded. Immunosuppressive regimens were based on induction therapy with basiliximab followed by triple therapy using tacrolimus, mycophenolate mofetil, and prednisolone. Daily intravenous infusion of 500 ml of dextran during the first five days after transplantation was used as a standard thrombose prophylaxis followed by per oral acetylsalicylic acid in dose of 75 mg daily during the first year after transplantation.

Donor parameters including age, sex, body mass index (BMI), serum transaminases, serum sodium, intensive care unit (ICU) stay, cause of donor brain death, percentage of liver graft steatosis, and recipient paramaters including age, sex, BMI, diagnosis, Model for End-Stage Liver Disease (MELD) score, time on waiting list, posttransplant time in ICU, cold ischemia time (i.e., from start of cold organ perfusion in the donor until portal reperfusion in the recipient), warm ischemia time (i.e., from removal of the liver from ice until portal reperfusion), days of hospitalisation, serum transaminases, gamma glutamyl transferase (GGT), alkaline phosphatase (ALP), and bilirubin and creatinine levels (preoperative and postoperative days 1, 2, 3, 4, 5, 6, 7, 14, and 30) were collected. Early allograft dysfunction (defined as the presence of one or more of the following findings: bilirubin ≥10 mg/dl (≥171 *μ*mol/L) on day 7, INR ≥ 1.6, and alanine transaminase (ALT) or aspartate aminotransferase (AST) > 2,000 IU/L (>34 *μ*kat/L) within the first 7 days [13]) and primary graft nonfunction (need for retransplantation up to day 10 or death due to graft nonfunction) [14] were also recorded.

Episodes of acute rejection within 30 days after transplantation and biliary and vascular complications were evaluated. Biliary complications were assessed by the review of radiological examinations, patient journals, and our local transplant registry. Patients were divided into the following groups depending on type of biliary complications: anastomotic strictures, nonanastomotic strictures [15], and bile leakage, followed by ERCP or PTC intervention at least once. Bile leakage was diagnosed by cholangiography or bilious secretion and always considered as a primary complication regardless of simultaneous complication of any other kind. Biliary strictures were categorized depending on the time of onset, early if occurring within 1 years of transplantation, or late, if occurring more than 1 year after transplantation.

The study protocol was approved by the Local Ethics Committee for Clinical Studies, and all procedures were performed according to the Helsinki Declaration.

## 3. Statistical Analysis

Descriptive statistics were used to summarise donor and recipient characteristics. Data are presented as mean ± SD. Cross-tabulation, the chi-squared test or Fisher's exact test were performed for comparison of the independent variables. Nonparametric variables were evaluated using the Mann–Whitney *U*-test. Parametric variables were evaluated using the independent-samples Student's *T* test. Multivariate analysis was carried out using a logistic regression model to analysis of the risk factor of biliary stricture. Kaplan–Meier survival curve testing was used for graft and patient survivals. *p*-values of <0.05 were considered to be statistically significant. Statistical calculations were performed using SPSS 21 software (SPSS Inc. Chicago, CA, USA).

## 4. Results

During the study period, a total of 546 orthotopic liver transplantations were performed at the Department of Transplantation Surgery, Karolinska University Hospital in Stockholm. 113 patients were excluded according to the exclusion criteria. Of the 433 patients, 230 (53.1%) received livers preserved in HTK and 203 (46.9%) in UW solution. Mean follow-up was 44.6 ± 29 months (0–105 months) in the HTK group and 42.4 ± 26 months (0–104 months) in the UW group. Recipients and donors in both groups were managed similarly with regard to operative techniques and immunosuppression. Donor characteristics are presented in Table 1. The groups differed in terms of total volume of preservation solution used during donor operation, as well as in cold ischemia time and donors' gender distribution. There was no difference in terms of causes of donor brain death between the two groups (data not shown). Percentage of hepatic steatosis and grade of ischemia injury in zero biopsy (taken at the end of recipient operation before closure of the abdomen) were the same in both groups (*p*=0.59 and *p*=0.12, respectively).

With regard to recipient characteristics, there were no differences between the groups (Table 2). Nor were any differences between the groups observed in terms of intraoperative parameters (Table 3).

Analysis of perioperative blood chemistry tests (hemoglobin, leukocytes, platelets, and CRP) showed no differences between the groups. Potassium levels after reperfusion and at the end of surgery were higher in the UW group (4.4 ± 0.7 vs 3.8 ± 0.6 mEq/L (*p* < 0.01) and 4.3 ± 0.6 vs 4.1 ± 0.5 mEq/L, respectively, *p* < 0.02; UW vs HTK, respectively). No differences were observed in post-transplant liver enzymes and serum creatinin (data not shown). There were no differences between the groups in terms of immunosuppressive regimens or used thrombosis prophylaxis.

There were no differences between the groups with regard to primary graft dysfunction and nonfunction (Table 4). Retransplantation was performed in seven patients (3%) in the HTK group and ten patients (4.9%) in the UW group (*p*=0.31). Two patients with primary nonfunction died (one following retransplantation), and two other patients were retransplanted with good graft function thereafter (Table 4).

With regard to postoperative complications, only incidence of biliary stricture differed between the groups (13.5% in the HTK group and 22.7% in the UW group; *p*=0.013). This difference was confirmed by multivariate analysis, and UW preservation was independently associated with the development of biliary stricture (CIT, solution type, and donor gender were entered in multivariate analysis; *p*=0.013, HR = 1.88, 95% CI: 1.1–3.1). Furthermore, period when biliary strictures were diagnosed was shorter in the UW group (the incidences of biliary stricture in the first year were 10% in the HTK group and 18.2% in the UW group; *p*=0.013) (Table 4).

**Figure 1:**
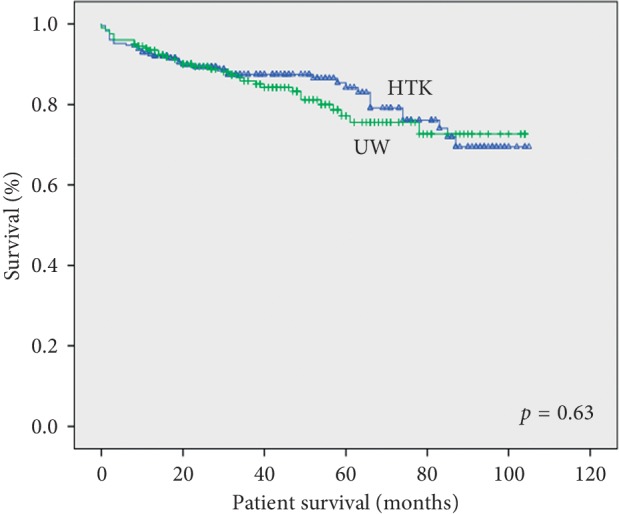
Kaplan–Meier patient survival for donor liver grafts preserved using HTK or UW.

**Figure 2:**
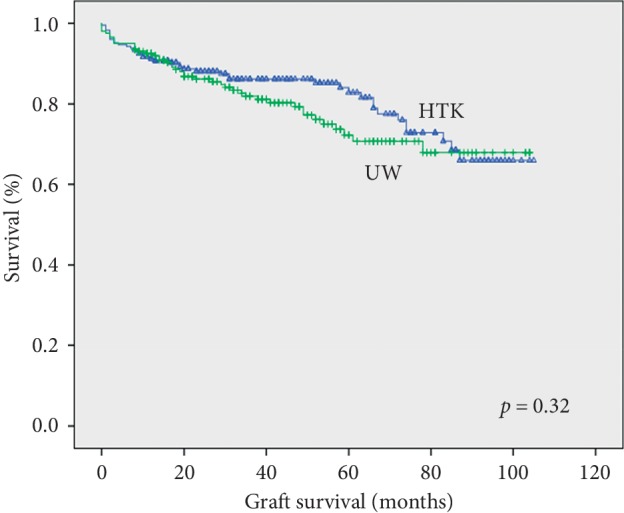
Kaplan–Meier graft survival for donor liver grafts preserved using HTK or UW.

There was no difference between the groups in terms of overall patient and graft survival (Figures 1 and 2). The overall and 1-, 6-, 12-, 36-, and 60-month patient survival rates were 83.5%, 98%, 94%, 92%, 87%, and 84% in the HTK group, and 82.8%, 98%, 96%, 93%, 85% and 77% in the UW group, respectively, *p*=0.63. There was no difference between the groups in terms of causes of recipient death (liver failure, sepsis, cardiovascular complications, original disease recurrence, and others) (data not shown). The overall, and 1-, 6-, 12-, 36-, and 60-month graft survival rates were 81.7%, 98%, 94%, 91%, 86%, and 82% in the HTK group, and 78.8%, 97%, 95%, 92%, 81%, and 72% in the UW group, respectively, *p*=0.32.

In the subgroup with cold ischemia time above 8 hours (115 patients in the HTK group and 139 patients in the UW group), the overall and 1-, 6-, 12-, 36-, and 60-month graft survival rates were 77.4% and 97%, 93%, 89%, 83%, 81% in the HTK group; 78.4% and 96%, 94%, 90%, 80%, 74% in the UW group, respectively, *p*=0.88. The overall and 1-, 6-, 12-, 36-, and 60-month patients survival rates were 81% and 97%, 94%, 92%, 86%, 84% in the HTK group; 82.7% and 98%, 96%, 92%, 84%, 79% in the UW group, respectively, *p*=0.78.

## 5. Discussion

The minimisation of organ preservation damage occurring during cold ischemia and reperfusion is critical in terms of outcome following solid organ transplantation. HTK solution has been adopted by many transplant centres as an alternative to UW preservation solution for routine preservation of the liver, kidney, and pancreas grafts. In this study, a comparison of UW and HTK solution for liver preservation was performed. One strength of our study is that although a formal randomisation was not performed, the donor livers were randomly perfused on a weekly basis served by two different transplant centres with either UW or HTK and from the same donor area. There was no difference between these two solutions in terms of clinical outcomes including graft and patient survival, primary nonfunction, primary dysfunction, and vascular complication. As expected, in our study, intraoperative potassium levels were higher in the UW group, though both preservation solutions maintained patient potassium levels within the normal range and of low clinical relevance. This can be related to high potassium content in UW solution, although there is no evidence that this is reflected in a higher serum potassium level in the recipient following reperfusion [16]. Despite routine use of Ringer acetate or blood to flush out UW solution from the preserved organ prior to reperfusion, a significant amount of UW solution remains in the organ, from where it is released into the recipient's blood stream after organ reperfusion.

In this study, biliary stricture rate was higher and period to the diagnosis was shorter in the UW group compared to the HTK group. Some studies showed that cold ischemia time and gender are risk factors for post-transplant biliary stricture [17, 18]. Despite these parameters differing between the groups in our series, in multivariate analysis, the only independent risk factor for biliary stricture was UW. Cold ischemia time is not an independent risk factor in our series. This might be due to the very small number of livers with CIT over 10 hours being transplanted at our centre. The differences in CIT between the two groups could be related to the significant proportion of liver grafts perfused with UW and brought in to our centre from other Scandinavian centres. It was postulated that the reduced viscosity of HTK solution, as compared to UW solution, has a protective effect against the development of biliary complications [18, 19]. However, the impact of HTK versus UW preservation solution on biliary complications remains unclear, as some centres report equivalent [20–22], increased [23, 24], or reduced [18, 19, 25] rates of biliary complication with HTK preservation solution in deceased donor liver allografts. Results from our study remain in line with results presented by Fridell et al., where significantly more recipients in the UW group required biliary imaging within one year of liver transplantation (51% in the HTK group, *n* = 371, vs 60% in the UW group, *n* = 327; *p*=0.01), and with the UW group having more biliary sludge (3.8% in the HTK group vs 11.3% in the UW group; *p*=0.001) [26]. A large registry analysis including 1,771 livers found that UW preservation was associated with more ischemic biliary lesions than HTK in univariate analysis, although in multivariate analysis, UW and HTK preservation was not independently associated with the development of ischemic biliary lesions [18]. Mangus et al. compared HTK vs UW in 698 liver transplant recipients (HTK 371, UW 327). They further categorised the liver allografts according to standard donor criteria (209/698, 30%) or extended donor criteria (489/698, 70%). There was no significant difference in graft survival between HTK and UW in any of the groups. They also found that HTK seems to have an advantage in terms of protection against biliary complications [19].

The effect of use of different type of preservation solution on post-transplant liver function tests is also unclear. Avolio et al. reported that AST on day 7 after transplantation in the HTK preservation solution group was lower than it was in the UW solution group [21]. Mangus et al. reported that initial median serum transaminase and bilirubin levels were higher in HTK solution-preserved livers but were similar by day 7 posttransplant [19]. In our study, there were no differences in liver function between the two groups at any time point.

In our series, PNF was seen in 1 (0.4%) patient in the HTK group, and 3 (1.5%) in the UW group. Incidence of PDF was seen in 20% in the HTK group, and in 17.7% in the UW group. There were no differences between the groups in terms of PNF and PDF, which was also presented in the systematic review by Feng et al. [1].

Canelo et al. reported on 123 liver transplants in which 63 were preserved using HTK and 71 using UW [27]. They also reported no differences between the HTK and UW groups with regard to patient and graft survival, ICU stay, and initial liver function values. In a prospective, observational, multicentre European study of 214 patients receiving HTK-preserved liver grafts, the primary dysfunction rate was 8.8% and primary nonfunction was 2.3%. One-year graft survival and patient survival were 83% and 80%, respectively [10].

In some studies, the cost of using HTK solution was found to be lower [28, 29] or equal [9] to use of UW solution. HTK solution costs are lower per litre. However, a larger volume of HTK is usually used during perfusion in the donor. The earliest studies of HTK preservation in clinical liver transplantation reported a use of large volumes of flush, with more than 10 L of HTK used compared to 4 L of UW solution [8, 9]. In our case series, the HTK-preserved livers received an average of only 1 L more preservation solution than the UW-preserved livers (HTK 5 L vs UW 4 L). Our results suggest that large volume infusion of HTK solution is not necessary in order to achieve safe organ preservation. Similar data were obtained by Mangus et al. where HTK-preserved livers received an average of only 0.6 L more preservation solution than the UW-preserved livers [28].

Stewart et al. presented a report from the UNOS database on the impact of HTK (*n* = 4755) vs. UW (*n* = 12673) preservation solutions on graft survival. They showed that HTK preservation was associated with an increased risk of graft loss, especially with DCD allograft and especially with cold ischemia time exceeding 8 h [11]. In the study presented, graft and patient survival rates were affected in both the groups when cold ischemia time exceeded 8 hours. This was somewhat more pronounced in the HTK group, though without being statistically significant.

In our study, there were no differences in terms of graft and patient survival between the HTK and UW solution groups. The potential effect of the preservation solution used could be minimised by the fact that a very few livers with CIT over 10 hours are transplanted at our centre.

A published registry study suggested that HTK was an independent risk factor for graft loss [12]; however, a systematic literature review and meta-analysis suggested that HTK solutions have similar clinical efficacy in terms of organ preservation during cold ischemia and the reperfusion phase compared with the UW solution [30].

In conclusion, our study shows that the use of UW and HTK solutions for liver preservation has no impact on graft or patient survival. However, biliary strictures were significantly more common and occurred earlier following transplantation in patients who received livers preserved in UW solution.

## Figures and Tables

**Table 1:** Donor characteristics for donor liver grafts preserved using HTK or UW.

	HTK (*n* = 230)	UW (*n* = 203)	*p*
Age (years)	53 ± 16.7	52 ± 16.4	0.64
Gender			**0.002** ^*∗*^
Male	103 (55.2%)	121 (40.4%)	
Female	127 (44.8%)	82 (59.6%)	
BMI (kg/m^2^)	25.6 ± 4.7	25.5 ± 4.4	0.81
Cold ischemia time (minutes)	496 ± 121	532 ± 113	**0.002** ^*∗*^
Warm ischemia time (minutes)	43.6 ± 14	46.8 ± 17.8	0.07
ICU time (days)	3.2 ± 4.4	3.2 ± 3	0.10
ALT (*μ*kat/L)	1 ± 1.9	1.1 ± 1.9	0.83
AST (*μ*kat/L)	1.1 ± 1.2	1.55 ± 2.7	0.94
BIL (*μ*mol/L)	16 ± 12	15 ± 13	0.19
Na (mmol/L)	147 ± 10.3	147 ± 13.9	0.76
Creatinine (*μ*mol/L)	93 (8–660)	103 (31–574)	0.20
Graft weight (g)	1,709 ± 429	1,614 ± 387	0.40
Total volume of solution (L)	5,393 (2,100–9,000)	4,138 (2,000–9,000)	**<0.01** ^*∗*^

HTK: histidine tryptophan ketoglutarate; UW: the University of Wisconsin; ALT: alanine aminotransferase; AST: aspartate aminotransferase; BIL: bilirubin; BMI: body mass index; ICU: intensive care unit. ^*∗*^*p* < 0.05, mean ± SD, median (min-max), *n* (%).

**Table 2:** Recipient characteristics for donor liver grafts preserved using HTK or UW.

	HTK (*n* = 230)	UW (*n* = 203)	*p*
Age (years)	51.4 ± 12.4	49.6 ± 13.5	0.20
Gender			0.53
Male	151 (65.7%)	139 (68.5%)	
Female	79 (34.3%)	64 (31.5%)	
BMI (kg/m^2^)	25.7 ± 4.4	25.9 ± 4.2	0.55
Diagnosis (%)			0.44
Alcohol	8.3%	4.4%	
HCC	23.5%	21.2%	
Cholestasis	25.2%	29.1%	
Hepatitis	19.1%	19.7%	
FAP	11.3%	7.9%	
Cryptogenic	2.6%	4.4%	
Acute failure	4.3%	4.9%	
Other	5.7%	8.4%	
MELD score	14.3 ± 7.9	14.5 ± 7.9	0.74
ICU time (days)	2.3 ± 3.4 (1–27)	2.4 ± 3.7 (1–15)	0.10
Days of hospitalisation (days)	18.3 ± 7.7 (8–51)	19.9 ± 9.3 (9–56)	0.06
Days on waiting list (days)	102 ± 98 (1–577)	99 ± 99 (1–513)	0.56

HTK: histidine tryptophan ketoglutarate; UW: the University of Wisconsin; BMI: body mass index; ICU: intensive care unit; MELD: model for end-stage liver disease; *n*: number; mean ± SD, median (min-max), *n* (%).

**Table 3:** Intraoperative finding of recipient operation for donor liver grafts preserved using HTK or UW.

	HTK (*n* = 230)	UW (*n* = 203)	*p*
Operation time (minutes)	428 ± 113	444 ± 128	0.18
Total bleeding (ml)	2,500 (125–40,500)	3,000 (150–50,000)	0.22
Intraoperative total blood transfusion (U)	4 (0–82)	5 (0–69)	0.71
Arterial flow (ml/minute)	372 ± 191	349 ± 171	0.08
Portal flow (ml/minute)	1,879 ± 841	1,848 ± 775	0.96
Number of arteries (%)			0.30
Simple	73%	68.5%	
Double	22.6%	28.6%	
Triple	4.3%	3%	
Venous by-pass (%)	18.7%	16.7%	0.59
Hepatic vein reconstruction (%)			0.20
Hepato-hepatic	73.9%	79.8%	
Side-to-side	6.1%	3%	
End-to-end	20%	17.2%	
Bile duct reconstruction (%)			0.64
Duct-to-duct	78.7%	76.8%	
Duct-to-enterostomy	21.3%	23.2%	
Bile duct stent tube (%)	40.4%	39.4%	0.82
Type of stent tube (% of tubes)			0.74
Internal	33.3%	31.2%	
T-tube	18.3%	15%	
External baby feeding	48.4%	53.8%	

HTK: histidine tryptophan ketoglutarate; UW: the University of Wisconsin; *n*: number; mean ± SD, median (min-max).

**Table 4:** Postoperative complications for donor liver grafts preserved using HTK or UW.

	HTK (*n* = 230)	UW (*n* = 203)	*p*
Postop reop due to bleeding (%)	7.1%	10.4%	0.22
Biliary stricture (%)	13.5%	22.7%	**0.013** ^*∗*^
Biliary stricture in first year	10%	18.2%	**0.013** ^*∗*^
Type of biliary stricture			0.61
Only anastomotic stricture	58.1%	52.2%	
Nonanastomotic stricture	41.9%	47.8%	
Bile leakage	7.8%	8.9%	0.69
Arterial complication	4 (1.7%)	4 (2%)	0.85
Portal complication	1 (0.5%)	2 (1%)	0.60
Primary nonfunction	1 (0.4%)	3 (1.5%)	0.34
Early allograft dysfunction (%)	20.4%	17.7%	0.47
Acute rejection first month (%) (biopsy-proven)	17%	16.7%	0.95

HTK: histidine tryptophan ketoglutarate; UW: the University of Wisconsin; *n*: number . ^*∗*^*p* < 0.05, *n* (%).

## Data Availability

The retrospective data used to support the findings of this study are included within the article.

## Abbreviations

ALP: Alkaline phosphatase
ALT: Alanine transaminase
AST: Aspartate aminotransferase
BMI: Body mass index
DDLT: Deceased donor liver transplantation
GGT: Gamma glutamyl transferase
HTK: Histidine tryptophan ketoglutarate
ICU: Intensive care unit
MELD: Model for end-stage liver disease
UW: The University of Wisconsin.
